# Dose-painting multicenter phase III trial in newly diagnosed glioblastoma: the SPECTRO-GLIO trial comparing arm A standard radiochemotherapy to arm B radiochemotherapy with simultaneous integrated boost guided by MR spectroscopic imaging

**DOI:** 10.1186/s12885-019-5317-x

**Published:** 2019-02-21

**Authors:** Anne Laprie, Soléakhéna Ken, Thomas Filleron, Vincent Lubrano, Laure Vieillevigne, Fatima Tensaouti, Isabelle Catalaa, Sergio Boetto, Jonathan Khalifa, Justine Attal, Guillaume Peyraga, Carlos Gomez-Roca, Emmanuelle Uro-Coste, Georges Noel, Gilles Truc, Marie-Pierre Sunyach, Nicolas Magné, Marie Charissoux, Stéphane Supiot, Valérie Bernier, Muriel Mounier, Muriel Poublanc, Amandine Fabre, Jean-Pierre Delord, Elizabeth Cohen-Jonathan Moyal

**Affiliations:** 1 Radiation Oncology Department, Institut Claudius Regaud- Institut Universitaire du Cancer de Toulouse-Oncopole, Toulouse, France; 2 ToNIC, Toulouse NeuroImaging Center, Université de Toulouse, INSERM, UPS, Toulouse, France; 3 Department of Engineering and Medical Physics, Institut Claudius Regaud- Institut Universitaire du Cancer de Toulouse-OncopoleCancer de Toulouse-Oncopole, Toulouse, France; 4 Biostatistics Unit, Institut Claudius Regaud- Institut Universitaire du Cancer de Toulouse-Oncopole, Toulouse, France; 5 Neurosurgery Department, Centre Hospitalier Universitaire de Toulouse, Toulouse, France; 6 Neuroimaging Department, Centre Hospitalier Universitaire de Toulouse, Toulouse, France; 7 Medical Oncology Department, Institut Claudius Regaud- Institut Universitaire du Cancer de Toulouse-Oncopole, Toulouse, France; 8 Pathology department, Centre Hospitalier Universitaire de Toulouse, Toulouse, France; 9 Radiation Oncology Department, Centre Paul Strauss, Strasbourg, France; 10 Radiation Oncology Department Centre Georges-François Leclerc, Dijon, France; 11 Radiation Oncology Department- Centre Léon Bérard, Lyon, France; 12 Radiation Oncology Department, Institut de Cancérologie de la Loire, Saint-Priest en Jarez, France; 13 Radiation Oncology Department - Centre Val d’aurelle, Montpellier, France; 14 Radiation Oncology Department, Institut de Cancerologie de l’Ouest, Nantes st Herblain, France; 15 Radiation Oncology Department, Institut de cancérologie de Lorraine centre Alexis Vautrin, Nancy, France; 16 Clinical Research Department, Institut Claudius Regaud, Institut Universitaire du Cancer de Toulouse-Oncopole, Toulouse, France; 17 INSERM UMR1037, Cancer Research Center of Toulouse, Oncopole, Toulouse, France

**Keywords:** Glioblastoma, Dose-painting, Magnetic resonance spectroscopic imaging, Spectroscopy, Proton spectroscopy, Radiotherapy, Clinical trial, Phase III,online prospective quality control

## Abstract

**Background:**

Glioblastoma, a high-grade glial infiltrating tumor, is the most frequent malignant brain tumor in adults and carries a dismal prognosis. External beam radiotherapy (EBRT) increases overall survival but this is still low due to local relapses, mostly occurring in the irradiation field. As the ratio of spectra of choline/N acetyl aspartate> 2 (CNR2) on MR spectroscopic imaging has been described as predictive for the site of local relapse, we hypothesized that dose escalation on these regions would increase local control and hence global survival.

**Methods/design:**

In this multicenter prospective phase III trial for newly diagnosed glioblastoma, 220 patients having undergone biopsy or surgery are planned for randomization to two arms. Arm A is the Stupp protocol (EBRT 60 Gy on contrast enhancement + 2 cm margin with concomitant temozolomide (TMZ) and 6 months of TMZ maintenance); Arm B is the same treatment with an additional simultaneous integrated boost of intensity-modulated radiotherapy (IMRT) of 72Gy/2.4Gy delivered on the MR spectroscopic imaging metabolic volumes of CHO/NAA > 2 and contrast-enhancing lesions or resection cavity. Stratification is performed on surgical and MGMT status.

**Discussion:**

This is a dose-painting trial, i.e. delivery of heterogeneous dose guided by metabolic imaging. The principal endpoint is overall survival. An online prospective quality control of volumes and dose is performed in the experimental arm. The study will yield a large amount of longitudinal multimodal MR imaging data including planning CT, radiotherapy dosimetry, MR spectroscopic, diffusion and perfusion imaging.

**Trial registration:**

NCT01507506, registration date December 20, 2011.

## Background

Glioblastoma (GBM) is an infiltrating heterogeneous brain tumor characterized by high cellular proliferation, high cellular density and active angiogenesis associated with areas of necrosis. After surgery or biopsy, irradiation is indicated as it improves overall survival, although most patients present local relapse in the irradiation fields. Prognosis is therefore dismal with a median overall survival of 8 to 14 months [1]. The failure to achieve sustainable local control in this tumor emphasizes the need to develop innovative treatment strategies. An attractive approach is to define new RT target volumes that include active disease, which can be highlighted by functional imaging. A promising strategy to tackle this radioresistance and attempt to improve local control consists of irradiating the target volume heterogeneously, with focal increases in dose targeted at radioresistant clusters defined by metabolic imaging [2, 3]. This ‘dose-painting’ approach targets metabolic abnormalities that are not only prognostic indicators of aggressiveness but are also predictive of local relapse after treatment.

In this context, in vivo ^1^H magnetic-resonance spectroscopic imaging (MRSI) has shown significant promise [4–7]. It measures the concentration and spatial distribution of tissue metabolites like choline (Cho) and N-acetyl-aspartate (NAA) which are respectively membrane and neuronal markers. An elevated Cho/NAA ratio (CNR) indicates increased cellular proliferation and reduced neuron density, and is assumed to highlight a metabolically active part of the tumor in high-grade gliomas [5, 7]. This metabolic tool is a useful predictor of survival [8, 9] and relapse location in GBM patients [6].

Our team showed [4] that magnetic resonance spectrometric imaging (MRSI) is useful in depicting areas with a high potential for recurrence. In that study, 23 reviews were conducted for 9 patients studied in a phase I associating tipifarnib and radiotherapy. Patients underwent MRI and MRSI before treatment and every 2 months after RT until relapse. The MRSI data were categorized by the choline (Cho) / N-acetyl-aspartate (NAA) ratio (CNR) to measure spectroscopic abnormalities. The study, which correlated spectral and morphologic abnormalities prior to radiotherapy with the same data at the time of relapse for 1207 voxels, showed that the volumes of spectral anomalies were more limited than morphological abnormalities. In fact, before radiotherapy, the CNR > 2 regions accounted for 25% of contrast regions and 10% of regions of T2 hyperintensity (excluding contrasting volumes). The presence of metabolically active regions was predictive of the site of relapse after radiotherapy. Indeed, 75% of the contrast enhanced lesion (CE) + CNR2 regions before radiotherapy continued to be CNR2 at relapse, compared to 22% of voxels with a normal CNR before RT (*p* < 0.05).

Several recent studies have investigated different methods for increasing the dose delivered to glioblastomas. Although previous studies showed only the feasibility of escalating doses without benefit but without toxicity, the most recent findings found a benefit in selected populations.

In 2004, Cho et al. [10] compared the modalities of boost after conformational radiotherapy (RTC) (60 Gy / 2Gy) delivered at an average 1.4 months after the end of RTC; 14 patients received a boost in radiosurgery with a median fraction of 10.5 Gy (10–18) and 10 others received a boost in stereotactic fractionated radiotherapy for a median dose of 27.5 Gy (20–35) in 11 fractions. There was an improvement in survival rates with fewer complications in the fractional stereotactic boost group. In 2004 Sultanem et al. [11] published a study of irradiation in intensity modulation in 20 fractions: 60 Gy / 3Gy on the GTV and 40 Gy / 2Gy on the PTV (GTV + 1.5 cm). Tolerance was good but survival was not modified. In 2002, Nwokedi et al. [12] reported a retrospective study comparing 33 patients who received 60 Gy / 2Gy with 31 patients who received gamma-knife radiosurgery of 17.1 Gy on average (10–28); in the group receiving the boost, survival was 25 months versus 13 months in the other group. Tolerance was identical. Tanaka et al. [13] showed that 80 Gy boosts of conformal external radiotherapy on contrast enhancement could lead to an increase in survival. However, these results need to be confirmed since there was no identical comparison group but only an historical comparison group. The more robust study by Cardinale et al. published in 2006 [14] reported the results of the RTOG0023 multicenter trial on 76 patients who received 50 Gy per 2 Gy fractions and a weekly stereotactic boost of 5 or 7 Gy, resulting in a dose equivalent of 70 or 78 Gy in 6 weeks. The treatment was feasible and well tolerated with a benefit only for patients with macroscopically complete resection. Of the 65 patients suspected of progression, half were re-operated: 15% had only necrosis and 85% had tumor necrosis.

These interesting dose-increase studies in newly diagnosed glioblastomas showing positive effects on survival did included neither concomitant temozolomide nor metabolic imaging to guide boost delivery, nor a concomitant daily boost included in the initial radiotherapy treatment. This prompted us to test concomitant radiochemotherapy with an integrated boost targeted at metabolic predictive abnormalities. We calculated the equivalent integrated boost of 72 Gy/2.4 Gy corresponding to the sequential boosts that had yielded survival improvement.

## Material and method

### Study design (Fig. 1)

The trial is a multicenter two-armed randomized phase III study

**Fig. 1:**
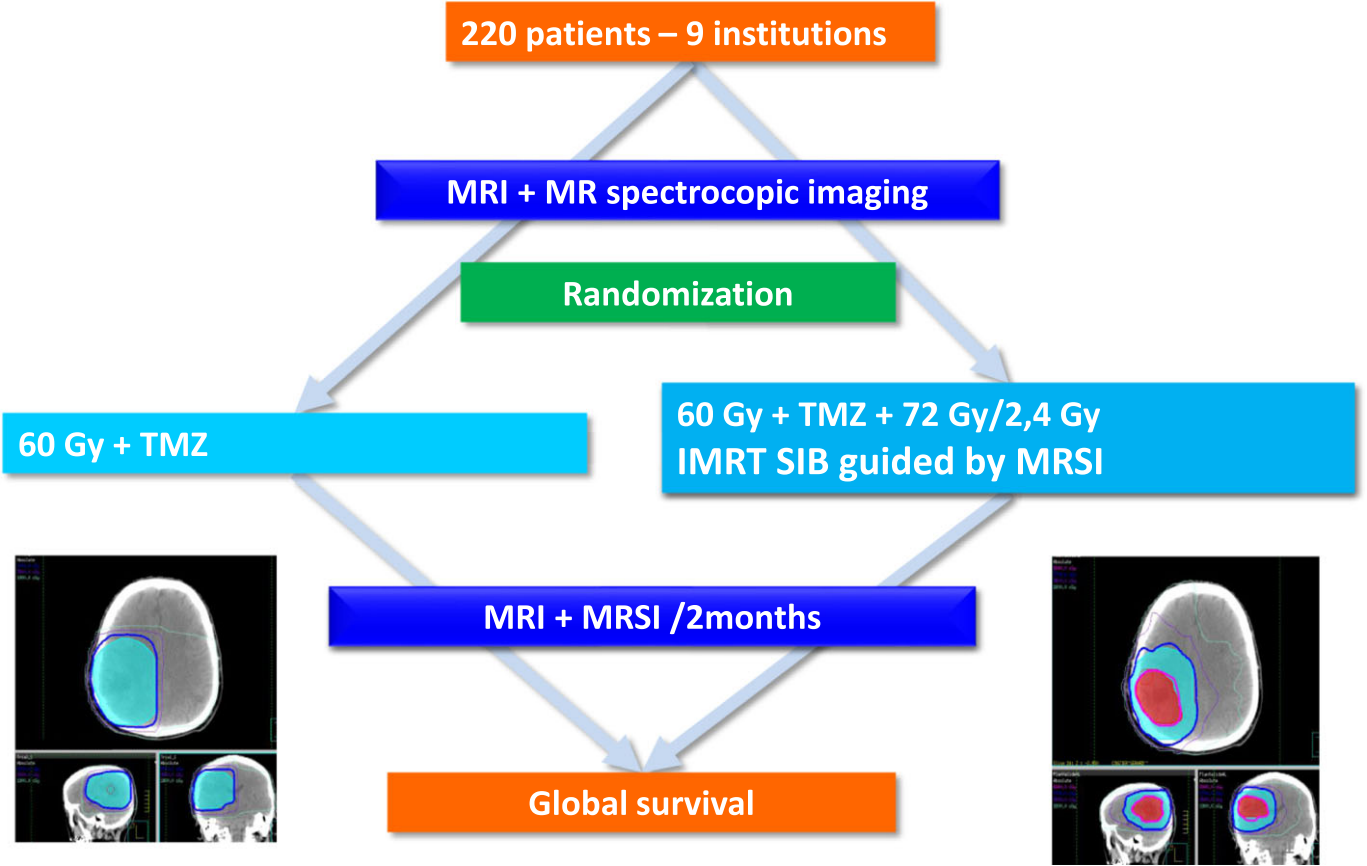
Study design

.

Patients fulfilling the inclusion criteria are randomized with computer-generated random numbers. Stratification is performed on surgical and MGMT status. The two arms are:

#### Arm a: Standard arm

3D conformal radiotherapy or IMRT delivering 60 Gy per fraction of 2 Gy in 30 fractions on contrast-enhancing lesions or tumor bed + 2 cm, with concomitant TMZ and 6 months maintenance of TMZ.

#### Arm B: Experimental arm

IMRT delivering 60 Gy per fraction of 2 Gy in 30 fractions on contrast-enhancing lesions or tumor bed + 2 cm, and 72GY/2.4 Gy with a simultaneous integrated boost guided at MRSI CHO/NAA > 2 + 10 mm and tumor bed + 3 mm with concomitant TMZ and 6 months maintenance of TMZ.

### Study objectives

Primary objective is overall survival. Secondary objectives are as follows: event-free survival; secondary effects of dose intensification, particularly intracranial hypertension, late effects such as radiation necrosis, increase in epileptic seizures; evaluation of individual, imaging and biologic markers associated with overall survival, event-free survival and sites of local relapse.

#### Patient selection: Inclusion criteria

- Age > 18 years
- PS ≤2
- Glioblastoma
- Biopsy or surgery
- Methylation status of MGMT gene
- In the event of surgery, the patient must have undergone an early MRI or CT scan to assess the presence of macroscopic residue.
- Surgery or biopsy performed in a maximum of 45 days before the first radiotherapy fraction. Randomization must be performed in the 32 days after surgery /biopsy to allow centralized contouring, dosimetry calculation and online prospective quality control.
- Written informed consent

#### Patient selection: exclusion criteria

- Impossibility to analyze MRSI metabolic maps, especially in the event of massive post-surgery hemorrhage inducing artefacts.
- Multifocal glioblastoma
- Leptomeningeal metastasis
- Epileptic attack despite anticonvulsant treatment
- Previous chemo- or radiotherapy to treat glioblastoma
- Abnormalities of blood cell count if neutrophils < 1500/mm^3^ (1.5 × 10^9^/l) and platelets < 100,000/mm^3^ (100 × 10^9^/l)
- Renal insufficiency
- Refusal to participate
- Previous re-irradiation or previous radiosurgery
- Previous treatment with interstitial radioactive seeds
- Carcinoma known < 5 years ago (excluding carcinoma in situ of the cervix, basal cell

carcinoma, squamous cell carcinoma of the skin) requiring immediate treatment interfering with study therapy.

- Pregnancy or lactation.

- Participation in another clinical trial

- Contraindications for MRI examination (e.g. claustrophobia, pacemaker)
- Disorder precluding understanding of trial or informed consent
- Diameter > 5 cm
- Distance from chiasma to tumor bed or MR spectroscopic abnormalities 2 cm

### Imaging and radiotherapy requirements

#### Imaging requirements

In this multicenter spectroscopy trial, the reproducibility and reliability of the technique are based on criteria for placement of the volume of interest, post-processing of spectral acquisition, quantification, and creation of metabolic maps. Findings to date have only been monocenter [4, 7, 15]. To ensure reliable and comparable post-processing between all centers, in the absence of “universal” spectrometry-processing software, all examinations are carried out on MRIs equipped with the CSI 3D module from the same manufacturer on a 1.5 T MRI scan (Siemens, Erlangen, Germany). Before inclusion began, each center performed an MRI + ISRM examination for 4 patients with glioblastoma according to the modalities defined for this protocol and sent the data to the promoter. Before the start of the trial, the coordinating center evaluated the quality of the images and spectra acquired. A team from the coordinating center including the coordinating radiation oncologist, the imaging-radiotherapy engineer and a physicist visited each center prior to the trial. This allowed any necessary modifications or improvements to be made before the trial began.

#### Radiotherapy requirements

The participating centers are academic or private centers, they must be able to use IMRT. Each center must perform an internal quality control of the accelerator. Furthermore, the calibration of the beams used in this study (external quality control) must comply with the requirements laid down in the technical decision of the AFSSAPS, the French health and safety agency (published 2 March 2004, updated 27 July 2007).

### Selection and randomization (Fig. 2)

After screening by the referring radiation oncologist, patients first undergo a multimodal MRI including T1 without and with gadolinium, FLAIR, diffusion-weighted sequences (DWI), perfusion-weighted imaging sequences (PWI) and 3D CSI MR spectroscopic imaging. Participating centers use a dedicated web-based database (equal ESTRO) to send MRI and spectroscopy post-processing data to the coordinating center. The MR spectroscopic imaging of CNR2 is obtained after analysis of the MR spectroscopy (Fig. 3). The size of the tumor and CNR2 must be < 5 cm and the tumor bed and CNR2 must be > 2 cm from the chiasma. Patients are then randomized.

**Fig. 2:**
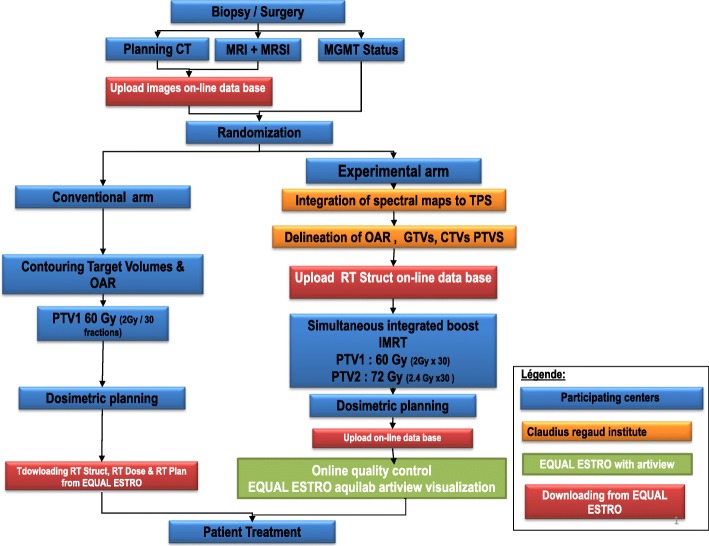
Study workflow

**Fig. 3:**
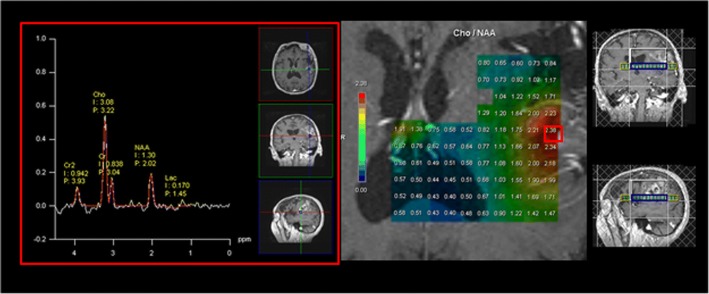
Example of an arm B patient:3A MRSI map showing abnormalities of Cho/NAA > 2

### Treatment planning

For arm B, all contours, including target volumes and organs at risk (OAR), are assessed by the coordinating center and sent to the participating center. Regarding technical difficulties for planning treatment, there are two main issues for including MRSI in a RT treatment planning system (TPS). Firstly, MRSI images obtained from MRI scanners do not conform to DICOM standards (DICOM 3.0) and are MR spectroscopic maps overlaid on corresponding anatomical MR images. These images, and unlike conventional MR images are not compatible for automatic image fusion with planning CT scans. Secondly, the escalation in radiation dose from the simultaneous integrated boost (SIB) should be carefully evaluated, especially for organs at risk (OAR).

We therefore performed a preliminary study to address these two issues and published [16] a method for incorporating metabolic maps into TPS, overcoming the absence of a DICOM 3.0 standard for MRSI, to guide the simultaneous integrated boost. In the same paper, we compared dosimetry plans of the standard 60 Gy treatment in 3D conformational radiotherapy (60-Gy 3D-CRT), 60 Gy in IMRT and treatment with the dose escalation of 72 Gy in SIB-IMRT. When comparing the dose received by OAR, there was no significant difference between 60-Gy 3D-CRT, 60 Gy IMRT and 72 Gy SIB-IMRT: i.e. there was no difference in the maximum dose to the optic chiasm and the mean dose to the normal brain. Compared to 60 Gy 3D-CRT, both 60-Gy IMRT and 72-Gy SIB-IMRT significantly lowered the dose to the brainstem. The method is used in this trial for including MR spectroscopic imaging of Cho/NAA > 2 when planning radiotherapy treatments.

### Dose prescription (Fig. 4 and 5)

Patients are treated in one of two arms. For the two treatment arms (definition on pre-RT MRI): GTV1: contrast enhancement on MRI pre-RT if biopsy, or if surgery on the operative bed and possible residual contrast on pre-RT MRI, CTV 1: GTV + 17 mm including T2 signal abnormalities, then corrected to remove bone and air.. PTV 1: CTV1 + 3 mm. No manual changes to PTV1 should be made.

**Fig. 4:**
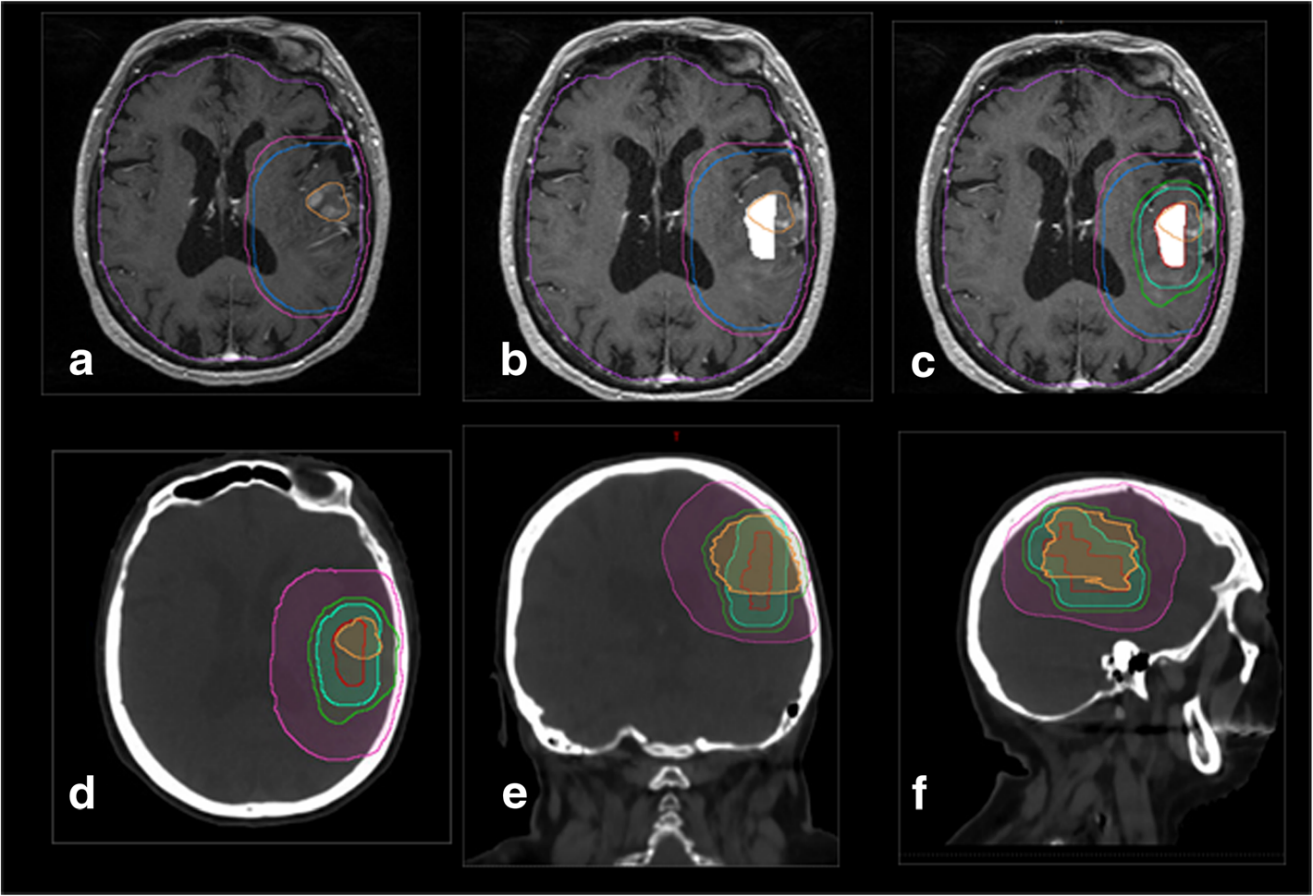
a-delineated GTV1(orange), CTV1 (blue), PTV1 (pink) on T1gado MRI, 4b MRSI map, 4Bc GTV1, CTV1, PTV1 GTV2(red), CTV2(cyan), PTV2 (green) 4d resulting volumes on axial view (**d**), frontal view (**e**) sagittal view (**f**)

**Fig. 5:**
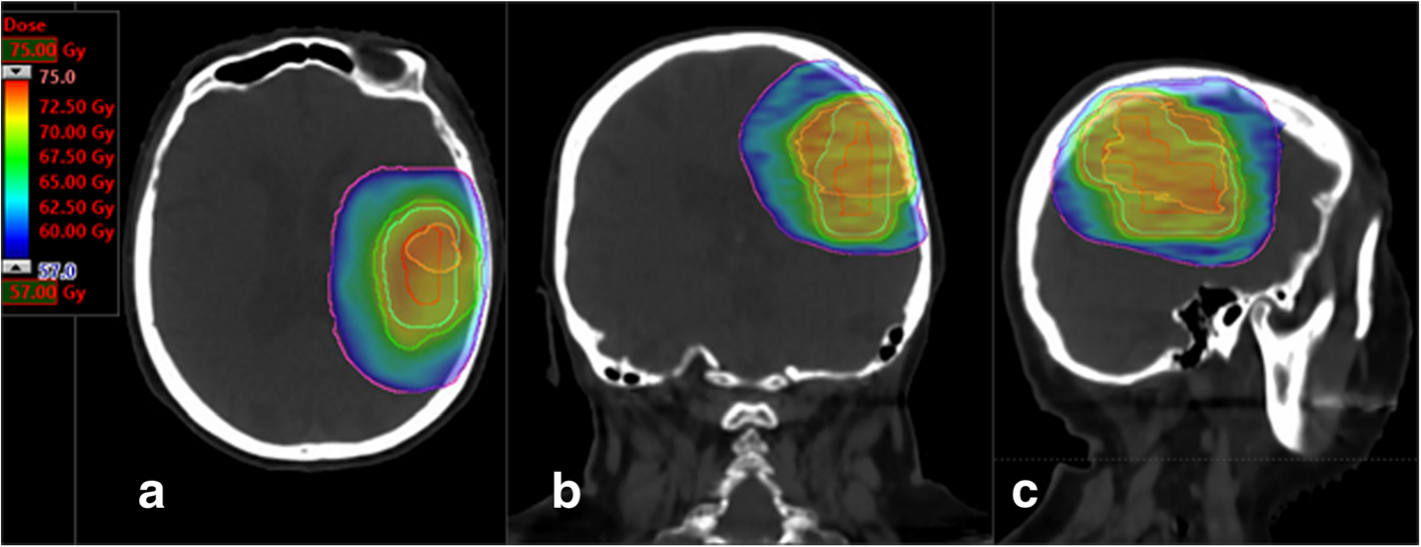
Corresponding dosimetry in **a**) axial slice, **b**) sagittal orientation, **c**) coronal orientation, GTVs, CTVs and PTVs are displayed with identical color lines as in Fig. 4. The isodoses are displayed in colorwash. In Blue 95% of 60Gy on PTV1, in orange 95% of 72Gy on PTV2

For Arm B, the GTV2, CTV2 and PTV2 are defined in addition to the following criteria: GTV2: Region defined in magnetic resonance spectrometry as the region with high relapse potential presents a signal finding Cho / NAA > 2 CTV2: GTV2 + 7 mm + GTV1 by correcting it to eliminate bone, air or other structure that cannot be extended. If CTV2 (presence of spectral abnormalities) is not included in CTV1, then CTV1 is expanded to include CTV2.

PTV2: CTV2 + 3 mm. No manual changes to PTV2 should be made. Delineated organs at risk are optic nerve, brainstem, optic chiasm, posterior chamber of eye, anterior chamber of eye, supra-tentorial brain.

PTV 2 receives a daily dose of 2.4 Gy for a cumulative dose of 72 Gy. Only irradiation with concomitant boost (SIB = simultaneous integrated boost) is permitted so sequential irradiation is not allowed. Irradiation is delivered in static, dynamic or arc intensity-modulated RT on linear accelerator or on tomotherapy. In each arm, treatment is associated with TMZ for 6 months after radiotherapy according to the standard Stupp protocol.

### Dose criteria for target volumes 

The PTV prescribing criteria must follow the recommendations of ICRU 83:

- 95% of the PTV should receive at least 95% of the dose
- 98% of the PTV should receive at least 90% of the dose
- 3% of the PTV should not receive more than 107% of the dose
- prescribed dose at the median of the PTV. In treatment arm B (with integrated boost), the prescribed doses are 60 Gy for PTV1 and 72 Gy for PTV2. Dose criteria for risk organs dose-volume histograms are generated for PTV, brain PTV, visual structures and brainstem.

During radiotherapy, temozolomide (TMZ) is administered orally at a dose of 75 mg / m2 / day, from the first day to the last day of radiation therapy, including Saturdays, Sundays and public holidays [17]. At the end of radiotherapy, the treatment with TMZ is stopped and then begins 4 weeks after the end of the radiotherapy according to the following modalities: temozolomide (TemodalR): Orally. - 150 mg / m2 / day for 5 days for the first cycle (from D1 to D5) - resumed at D 28 (D28 to D32) and then every 28 days at 200 mg / m2 / day for 5 days if the hematological tolerance of the first cycle was good (in total 6 cycles of 5 days of adjuvant TMZ).

### Imaging data acquisition

Before inclusion and at each follow-up, patients undergo an MRI scan that includes: anatomical T1-weighted sequences, before and after contrast injection, FLAIR, T2, 3D MRSI, perfusion weighted imaging (PWI) with dynamic susceptibility contrast (DSC) and diffusion-weighted imaging (DWI). All scans in each center are performed on a Siemens 1.5-T MRI scanner (Siemens, Erlangen, Germany) with a standard 12-channel head coil. Parallel imaging acquisition is used for each patient, based on the GRAPPA (Generalized autocalibrating partially parallel acquisition) reconstruction algorithm, with an acceleration factor of 2. The anatomical MRI protocol includes: acquisition of 3 mm-thick axial images (turbo spin-echo T2-weighted imaging (TR/TE = 4200/97 ms), (FLAIR: TR/TI/TE = 6500/2400/121 ms)) with a field of view (FOV) of 172 × 230 mm^2^ and a matrix size of 256 × 192, resulting in a voxel size of 0.9 × 0.9 × 3 mm^3^; and acquisition of 1 mm-thick 3D T1-weighted images (T1) before and after injection of a standard dose of 15 ml of gadolinium-based contrast agent (Gadobenate dimeglumine, MultiHance®) (T1-Gd: TR/TE = 11/5.2 ms, FOV = 256 × 224, matrix size = 256 × 224, resulting in a voxel size of 1 × 1 × 1 mm^3^).

3D-chemical-shift imaging (3D-CSI) for MRSI acquisition consists of three phase-encoded gradients prior to read-out, resulting in a scan time of 8 min. MRSI acquisition consists of a spin-echo-based sequence with the following parameters: TR/TE = 1500 ms/135 ms for lactate detection, and four excitations. FOV is set by default at 100 × 100 mm^2^ for a CSI matrix of 16 × 16, with eight slices of 25.0 mm thickness, resulting in a voxel resolution of 6.26 × 6.25 × 25.0 mm^3^, i.e. 1 cm^3^. Adjustments on the 3D-CSI box are performed when needed to cover entirely or the majority of abnormalities and when possible normal-appearing tissue, while avoiding regions that could corrupt the spectra such as bone and subcutaneous lipids. Saturation bands are also positioned around the volume of interest (VOI) to suppress signals from excited regions outside the VOI, and to provide good in vivo fat suppression. Manual shimming is performed to reach a line width < 15 Hz.

For DSC-MRI, a series of 34 volumes of 39 slices are acquired at 1.09-s intervals, with a gradient-echo echo-planar imaging sequence (TR/TE = 4720/47 ms, FOV = 230 × 230 mm^2^, matrix size = 128 × 128, flip angle = 90°), during the first pass of a standard dose (0.1 mmol/kg) bolus of gadolinium. Gadolinium is injected intravenously using a power injector at a rate of 5 mL/s, immediately followed by a saline injection. DWI is performed with a single-shot, spin-echo, echo-planar imaging sequence in the axial plane (TR/TE = 8300/91 ms at b = 0 and b = 1000 s/mm2, 25–40 sections, 2-mm section thickness, FOV = 230 × 230 mm^2^, matrix size = 192 × 192). DWI is acquired in three orthogonal directions. CT simulation images for RT planning of all patients are acquired in helical mode with a voxel resolution of 0,98 × 0.98X 2.5 mm^3^.

### Spectroscopic data processing

The spectroscopic processing protocol consists of water subtraction, low-pass filtering, frequency shift correction, baseline correction, phase correction and curve-fitting in the frequency domain. These steps of spectra processing are performed with the Siemens Syngo MR B17 spectroscopy application (Erlangen, Germany).

#### Quality control

A dummy run study is performed in both arms, both for MR spectroscopic acquisition and for arm A contouring + dosimetry as well as dosimetry for arm B.

Online contouring for arm B.

Online control quality of dosimetry for arm B.

This procedure is described in Fig. 2 and the delay for starting radiotherapy the latest at 42 days after biopsy or surgery must be respected. Then the dosimetry is performed in the participating center and arm B provisional dosimetries undergo prospective online quality control performed by an independent physicist and by an independent radiation oncologist reviewer.

#### Treatment schedule

The treatment must start within 45 days after biopsy or surgery. The fractions should be delivered within 42 days (6 weeks) and the total duration of treatment must not exceed 48 days. A bi-fractionation (2 fractions delivered the same day at more than 6 h interval) is allowed a maximum of three times during the treatment and not more than once a week. The patient receives 5 sessions maximum per week, bi-fractionations included. Treatment interruptions must be clearly recorded in the treatment record as well as the reasons for the interruptions. There must be no more than 3 consecutive interruption working days and a maximum of 6 days in total. Interruptions are tolerated only for medical reasons due to severe side effects or other concomitant diseases, but not for social or logistical reasons.

### Follow-up

#### Clinical follow-up

Patients undergo medical examination every week during radiotherapy and every 21 days during maintenance temozolomide.

#### Imaging procedures

MRI (including T1 without and with gadolinium enhancement, T2, FLAIR, PWI(DSC), DWI and MRSI (3D-CSI) examinations are carried out as follows:

- 1st examination 3 months after the end of radiotherapy in order to have the maximum effect of radiotherapy and to avoid aspects of pseudo-progression [18], then every 2 months. In the interest of the patient, if the patient shows suspicious clinical signs within 3 months after the end of radiotherapy, MRI and MRSI control are performed earlier. Due to the risk of pseudo-progression, a new MRI + MRSI is performed 1 month later in order to verify (to check) the reality of the progression (for any image of progression or new contrast in radiation field during the first MRI of control) [18]. In the event of stability or regression, the patient is considered as non-relapsing.

#### Definition of event

Relapse is defined with the RANO criterias [19]. In the event of relapse, treatment is decided upon by the investigator.

Adverse events and other unintended effects of trial interventions or trial conduct this events are collected by the research technicians, reported and an external security toxicity audit is performed every year.

### Statistical analysis

The main objective is to increase 2-year overall survival from 25% (standard arm “radiotherapy plus temozolomide”) to 40% in the experimental arm. This hypothesis corresponds to detecting a hazard ratio of 0.66. A total of 186 deaths are necessary for 80% power to detect this difference if it is true using a two–sided logrank test at the 5% level of significance and a 1:1 randomization. Based on an estimated accrual rate of approximately 70 patients per year and a fixed follow-up of 3 years, 220 patients need to be included. An interim analysis for efficacy will be performed after 93 events have been observed (Lan deMets O’Brien Fleming Boundaries).

## Discussion

The SPECTRO GLIO trial is one of the few ongoing dose-escalation or dose-painting trials in Europe [20–24]. It is based on the value of an advanced type of metabolic imaging to predict the site of relapse for glioblastoma after radiotherapy, i.e. CHO/NAA ratio > 2. MR spectroscopic imaging is used as a target. The use of SIB makes it possible to increase significantly the dose without increasing the dose to organs at risk [16].

A strength of the trial is the centralized analysis of MR spectroscopy, the centralized contouring for the experimental arm, and the online prospective quality control of dosimetry in the experimental arm. As described for several trials [25, 26] and in two recent metaanalysis [27, 28], we strongly believe that quality control and homogeneity of treatment are crucial to provide reliable results in this type of trial. By February 2018, 180 patients had been included and the intermediate analysis is planned after 93 events.

The issue of glioblastoma dose-painting is a shared interest as a comparable phase II trial with a 75 Gy boost targeted at CNR > 2 started in September 2017 in the US (NCT03137888) as a pilot study in 36 patients with the use of whole brain MR spectroscopy. The SPECTRO GLIO trial includes a large amount of imaging data and dosimetry information: planning CT, all contours of organs at risks and target volumes, longitudinal T1, FLAIR, diffusion and perfusion MRI, as well as 3D MR Spectroscopic imaging. This large longitudinal set of data will provide useful information on the natural history of glioblastoma correlated with different levels of radiotherapy dose. We have already performed imaging studies on subsets of patients included in this trial [29, 30] and will carry out further studies on the whole cohort, as this may lead to new prognostic and predictive values for anatomic and metabolic imaging, particularly the predictive value of CHO/NAA and lactates/NAA [31] already published in small subsets of patients.

## Abbreviations

3D-CRT: Three-dimensional conformation radiation therapy
CE: contrast enhancement
CSI: chemical Shift imaging
DSC: Dynamic Susceptibility contrast
DWI: Diffusion Weighted Imaging
FLAIR: Fluid Attenuated Inversion Recovery
GBM: glioblastoma
IMRT: intensity Modulated Radiotherapy
MRI: magnetic resonance imaging
MRSI: Magnetic Resonance Spectroscopic Imaging
PWI: Perfusion Weighted Imaging
SIB: Simultaneous Integrated Boost
TMZ: Temozolomide
